# Exposure to any antenatal corticosteroids and outcomes in preterm infants by gestational age: prospective cohort study

**DOI:** 10.1136/bmj.j1039

**Published:** 2017-03-29

**Authors:** Colm P Travers, Reese H Clark, Alan R Spitzer, Abhik Das, Thomas J Garite, Waldemar A Carlo

**Affiliations:** 1 Division of Neonatology, University of Alabama at Birmingham, AL 35233, USA; 2 Center for Research, Education, and Quality, Pediatrix Medical Group and MEDNAX, Sunrise, FL, USA; 3 Social, Statistical and Environmental Sciences Unit, RTI International, Research Triangle Park, NC, USA; 4 Department of Obstetrics and Gynecology, University of California, Irvine, CA, USA

## Abstract

**Objective** To determine whether exposure to any antenatal corticosteroids is associated with a lower rate of death at each gestational age at which administration is currently recommended.

**Design** Prospective cohort study.

**Settings** 300 participating neonatal intensive care units of the Pediatrix Medical Group in the United States.

**Participants** 117 941 infants 23 0/7 to 34 6/7 weeks’ gestational age born between 1 January 2009 and 31 December 2013.

**Exposure** Any antenatal corticosteroids.

**Main outcomes measures** Death or major hospital morbidities analyzed by gestational age and exposure to antenatal corticosteroids with models adjusted for birth weight, sex, mode of delivery, and multiple births.

**Results** Infants exposed to antenatal corticosteroids (n=81 832) had a significantly lower rate of death before discharge at each gestation 29 weeks or less, 31 weeks, and 33-34 weeks compared with infants without exposure (range of adjusted odds ratios 0.32 to 0.55). The number needed to treat with antenatal corticosteroids to prevent one death before discharge increased from six at 23 and 24 weeks’ gestation to 798 at 34 weeks’ gestation. The rate of survival without major hospital morbidity was higher among infants exposed to antenatal corticosteroids at the lowest gestations. Infants exposed to antenatal corticosteroids had lower rates of severe intracranial hemorrhage or death, necrotizing enterocolitis stage 2 or above or death, and severe retinopathy of prematurity or death compared with infants without exposure at all gestations less than 30 weeks and most gestations for infants born at 30 weeks’ gestation or later.

**Conclusion** Among infants born from 23 to 34 weeks’ gestation, antenatal exposure to corticosteroids compared with no exposure was associated with lower mortality and morbidity at most gestations. The effect size of exposure to antenatal corticosteroids on mortality seems to be larger in infants born at the lowest gestations.

## Introduction

In meta-analyses of randomized controlled trials of antenatal corticosteroids analyzed by gestational age at birth, death was decreased in the subgroup of preterm infants born at 30-34 weeks’ gestation but not in the subgroup born before 30 weeks’ gestation, in part because of the small sample size.^1^ ^2^ These same meta-analyses also analyzed by gestational age at trial entry and found a lower rate of death among infants from 26 weeks’ gestation and higher. As current guidelines recommend the administration of antenatal corticosteroids to women at risk of preterm delivery from 24 0/7 to 34 6/7 weeks’ gestation and agree on considering administration from 23 0/7 to 23 6/7,^3^ ^4^ ^5^ further randomized controlled trials are unlikely to be conducted in this population. The current recommendations for infants at the lowest gestations are based on limited evidence from randomized controlled trials, but the extension of the recommendation to consider administration below 24 weeks is based on consensus and recent observational studies in selected populations.^3^ ^4^ ^5^ ^6^ ^7^ ^8^ ^9^ ^10^

As some of the benefits of antenatal corticosteroids differ by gestational age,^1^ ^2^ the objective of this study was to determine the presence and magnitude of the association between antenatal corticosteroids and outcomes at each gestational age for which this treatment is currently recommended. We hypothesized that in preterm infants with a gestational age of 23 0/7 to 34 6/7 weeks, exposure to antenatal corticosteroids compared with no exposure to antenatal corticosteroids would be associated with a lower rate of hospital mortality (primary outcome) and major morbidities at each gestational age.

## Methods

This study analyzed data collected prospectively at 300 participating neonatal intensive care units in the United States for the database of the Pediatrix Clinical Data Warehouse. This database includes clinical data acquired electronically from medical records of all infants admitted to neonatal intensive care units of the Pediatrix Medical Group. The Pediatrix Medical Group includes a range of both large and small neonatal intensive care units, academic and non-academic centers, and several children’s hospital centers in the US. This database has been previously well described and has been used to answer major research questions and for large collaborative quality improvement initiatives.^11^ ^12^ ^13^ We included infants from 23 0/7 to 34 6/7 weeks’ gestation born between 1 January 2009 and 31 December 2013. We prioritized best obstetric estimate of gestational age based on the mother’s last menstrual period and fetal ultrasonography over best neonatal estimates as determined on the basis of physical examination criteria, including the Ballard or Dubowitz examination. We excluded infants who were not delivered at one of the study hospitals, infants with major anomalies, and those who were transferred to another hospital, to ensure that all outcome data for infants in this study were complete. We did not impute missing data.

### Definitions

We considered infants whose mothers had received one or more doses of either betamethasone or dexamethasone to be exposed to any antenatal corticosteroids.^14^ Data on partial or complete courses and data on repeat courses of antenatal corticosteroids were not collected.^15^ We abstracted all outcomes prospectively from medical records by using standardized definitions. Data were collected until death or discharge, whichever occurred first. We defined severe intracranial hemorrhage (grade 3-4) by using Papile’s grading system and medical or surgical necrotizing enterocolitis (stage ≥2) by using the modified Bell’s criteria. Infants diagnosed as having suspected necrotizing enterocolitis (stage 1) were not included. We defined severe retinopathy of prematurity (stage ≥3 or treated with ablation/antivascular endothelial growth factor) by using the International Classification of Retinopathy of Prematurity and bronchopulmonary dysplasia by using the traditional definition of treatment with supplemental oxygen or respiratory support at 36 weeks’ postmenstrual age.^16^ We classified infants with one of more of the above conditions as having major hospital morbidity.

### Outcome measures

Many of the major outcomes in neonatology compete with death and can be assessed only in infants who survive the initial hospital course. Therefore, we reported these outcome measures with death as a competing composite outcome as well as independently of death as a non-composite outcome.

### Statistical analysis

We explored the association between antenatal corticosteroids and outcomes separately within each gestational age group. The primary outcome measure was death before discharge from hospital. All secondary outcome measures were pre-specified and included both composite outcomes combined with death and non-composite outcomes. We used Fisher’s exact test to describe differences in categorical variables. We used analysis of variance for normally distributed continuous variables and the Kruskal-Wallis test for continuous skewed variables. We did logistic regression analyses adjusted for factors present at birth previously associated with differences in mortality, including birth weight, sex, mode of delivery, and multiple births.^17^ ^18^ ^19^ We calculated the number needed to treat to prevent one death by using the standard method of unadjusted absolute risk reduction at each gestational age.

We also did forward stepwise logistic regressions for other factors present at birth associated with neonatal outcomes, including race/ethnicity, small for gestational age birth (<10th centile), maternal age, diabetes, pre-eclampsia, hypertension, prenatal care, rupture of membranes more than 24 hours before birth, and placental abruption. We defined a probability significance level to enter the model as 0.15 and a probability significance level to leave the model as 0.1. Within each gestational age, the factors listed above that were retained were different but the adjusted odds ratio was not changed significantly, so none of these factors was retained in the final model. We used SAS software version 9.2 for all statistical analyses. We estimated odds ratios and 95% confidence intervals for binary outcomes, with a two sided P value of less than 0.05 indicating statistical significance.

### Patient involvement

Patients/parents and advocacy groups were not involved in the development of the study hypothesis, recruitment, outcome measures, study design, or implementation. Patients/parents and advocacy groups were not involved in the interpretation of the results or in the preparation of the manuscript, and there are no plans to disseminate the results of the research directly to study participants, advocacy groups, or parent groups.

## Results

We included 117 941 infants, of whom 81 832 (69.4%) were exposed to antenatal corticosteroids (table 1). Rates of exposure to antenatal corticosteroids were lower among infants at the higher and the lower ends of the recommended gestational age range (fig 1). Mothers of infants who were exposed to antenatal corticosteroids were more likely to be white, to be delivered by cesarean section, and to have had prenatal care, rupture of membranes more than 24 hours before birth, pre-eclampsia, and hypertension. Infants who were exposed to antenatal corticosteroids were more likely to be small for gestational age and the product of multiple births (table 1).

**Table 1 tbl1:** Infant/maternal baseline characteristics by antenatal corticosteroid treatment. Values are numbers (percentages)

	Antenatal corticosteroids (n=81 832)	No antenatal corticosteroids (n=36 109)
**Mother**		
Race/ethnicity:		
Black	17 977 (22.0)	7975 (22.1)
White	41 876 (51.2)	16 180 (44.8)
Hispanic	14 392 (17.6)	7831 (21.7)
Prenatal care reported	78 280 (95.7)	33 195 (91.9)
Pre-eclampsia	12 051 (14.7)	3436 (9.5)
Hypertension	15 508 (19.0)	5349 (14.8)
Placental abruption	3726 (4.6)	2088 (5.8)
Diabetes	9229 (11.3)	3855 (10.7)
Rupture of membranes >24 h	14 129 (17.3)	2298 (6.4)
**Infant**		
Male sex	42 999 (52.5)	19 213 (53.2)
Multiple births	26 241 (32.1)	9149 (25.3)
Small for gestational age	9655 (11.8)	3113 (8.6)
Cesarean section	56 004 (68.4)	21 615 (59.9)

Cohort includes infants delivered between 1 January 2009 and 31 December 2013 with gestational age 23 0/7 to 34 6/7 weeks.

All parameters differed significantly between groups (P<0.05).

**Figure f1:**
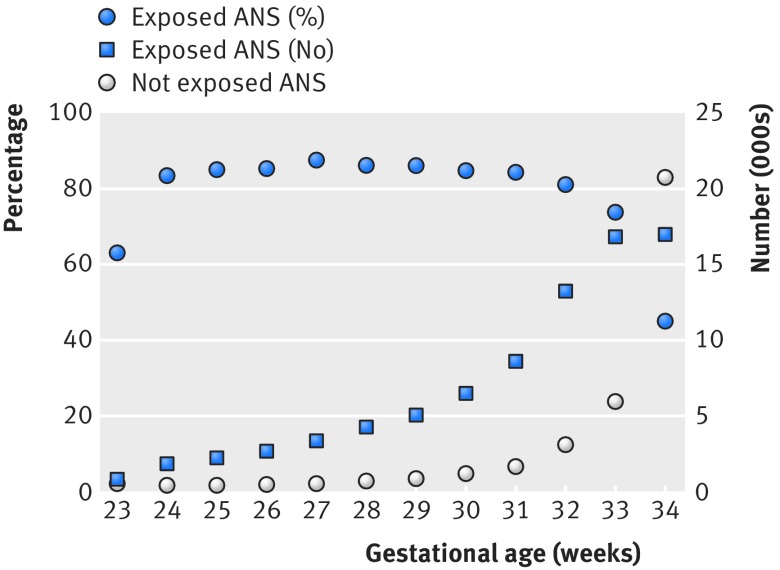
**Fig 1** Exposure to antenatal corticosteroids (ANS) by gestational age. Rates of exposure to antenatal corticosteroids were lower among infants at higher and lower ends of recommended gestational age range

Among infants exposed to antenatal corticosteroids, the mortality rate was lower than among those without exposure at each gestational age from 23 to 29 weeks and at 31, 33, and 34 weeks, an association that persisted after adjustment for covariates (table 2). The unadjusted number needed to treat to prevent one infant death increased exponentially with increasing gestational age, rising from six infants at 23 and 24 weeks’ gestation to 798 infants at 34 weeks’ gestation (fig 2). The adjusted odds ratios calculated using gestational age specific forward stepwise regression models did not differ in a meaningful way from the results of the gestational age specific models calculated using previously identified risk factors for mortality, and they are therefore not presented.

**Table 2 tbl2:** Mortality and composite outcomes of infants born 23 0/7 to 34 6/7 by gestational age and exposure to antenatal corticosteroids (ANS). Values are numbers (percentages) unless stated otherwise

	23 weeks	24 weeks	25 weeks	26 weeks	27 weeks	28 weeks	29 weeks	30 weeks	31 weeks	32 weeks	33 weeks	34 weeks
**Death before discharge**												
ANS	439/754 (58.2)	642/1781 (36.0)	432/2161 (20.0)	302/2602 (11.6)	213/3315 (6.4)	141/4237 (3.3)	90/5019 (1.8)	75/6466 (1.2)	45/8556 (0.5)	44/13 203 (0.3)	22/16 810 (0.1)	9/16 928 (0.1)
No ANS	331/447 (74.0)	182/352 (51.7)	114/381 (29.9)	77/444 (17.3)	51/468 (10.9)	46/685 (6.7)	30/813 (3.7)	22/1172 (1.9)	21/1591 (1.3)	18/3070 (0.6)	18/5954 (0.3)	37/20 732 (0.2)
AOR (95% CI)	0.47 (0.36 to 0.62)	0.51 (0.40 to 0.64)	0.52 (0.41 to 0.67)	0.55 (0.42 to 0.74)	0.50 (0.36 to 0.71)	0.48 (0.34 to 0.69)	0.44 (0.29 to 0.68)	0.66 (0.41 to 1.12)	0.42 (0.25 to 0.74)	0.61 (0.36 to 1.08)	0.43 (0.23 to 0.80)	0.32 (0.14 to 0.63)
**Severe intracranial hemorrhage or death**												
ANS	493/754 (65.4)	774/1781 (43.5)	607/2161 (28.1)	449/2602 (17.3)	329/3315 (9.9)	247/4237 (5.8)	162/5019 (3.2)	132/6466 (2.0)	95/8556 (1.1)	75/13 203 (0.6)	47/16 810 (0.3)	21/16 928 (0.1)
No ANS	364/447 (81.4)	214/352 (60.8)	144/381 (37.8)	128/444 (28.8)	90/468 (19.2)	70/685 (10.2)	52/813 (6.4)	46/1172 (3.9)	35/1591 (2.2)	28/3070 (0.9)	27/5954 (0.5)	47/20732 (0.2)
AOR (95% CI)	0.40 (0.29 to 0.53)	0.47 (0.37 to 0.60)	0.57 (0.45 to 0.72)	0.48 (0.38 to 0.60)	0.43 (0.33 to 0.57)	0.55 (0.41 to 0.73)	0.47 (0.34 to 0.66)	0.53 (0.38 to 0.76)	0.53 (0.36 to 0.80)	0.64 (0.42 to 1.01)	0.60 (0.37 to 0.97)	0.56 (0.33 to 0.94)
**Necrotizing enterocolitis ≥stage 2 or death**												
ANS	464/754 (61.5)	704/1781 (43.5)	536/2161 (24.8)	427/2602 (16.4)	322/3315 (9.7)	276/4237 (6.5)	225/5019 (4.5)	212/6466 (3.3)	175/8556 (2.0)	176/13 203 (1.3)	122/16 810 (0.7)	52/16 928 (0.3)
No ANS	342/447 (76.5)	198/352 (56.3)	127/381 (33.3)	99/444 (22.3)	76/468 (16.2)	69/685 (10.1)	53/813 (6.5)	57/1172 (4.9)	40/1591 (2.5)	56/3070 (1.8)	45/5954 (0.8)	97/20 732 (0.5)
AOR (95% CI)	0.47 (0.36 to 0.62)	0.48 (0.37 to 0.60)	0.58 (0.46 to 0.74)	0.62 (0.49 to 0.81)	0.52 (0.39 to 0.69)	0.62 (0.47 to 0.83)	0.64 (0.47 to 0.89)	0.66 (0.49 to 0.91)	0.85 (0.60 to 1.23)	0.71 (0.53 to 0.98)	0.93 (0.67 to 1.33)	0.67 (0.47 to 0.94)
**Severe retinopathy of prematurity or death**												
ANS	543/754 (72.0)	936/1781 (52.6)	704/2161 (32.6)	487/2602 (18.7)	298/3315 (9.0)	203/4237 (4.8)	133/5019 (2.6)	99/6466 (1.5)	63/8556 (0.7)	54/13 203 (0.4)	25/16 810 (0.1)	10/16 928 (0.1)
No ANS	367/447 (82.1)	228/352 (64.8)	161/381 (42.3)	114/444 (25.7)	67/468 (14.3)	56/685 (8.2)	36/813 (4.4)	27/1172 (2.3)	23/1591 (1.4)	19/3070 (0.6)	20/5954 (0.3)	39/20 732 (0.2)
AOR (95% CI)	0.55 (0.40 to 0.74)	0.55 (0.43 to 0.70)	0.57 (0.45 to 0.71)	0.58 (0.45 to 0.74)	0.52 (0.39 to 0.70)	0.53 (0.39 to 0.74)	0.56 (0.38 to 0.84)	0.68 (0.44 to 1.09)	0.55 (0.34 to 0.93)	0.67 (0.40 to 1.16)	0.43 (0.24 to 0.78)	0.33 (0.15 to 0.63)
**Bronchopulmonary dysplasia or death**												
ANS	660/754 (87.5)	1359/1781 (76.3)	1384/2161 (64.0)	1256/2602 (48.3)	1137/3315 (34.3)	969/4237 (22.9)	711/5019 (14.2)	608/6466 (9.4)	556/8556 (6.5)	635/13 203 (4.8)	511/16 810 (3.0)	366/16 928 (2.2)
No ANS	420/447 (94.0)	287/352 (81.5)	251/381 (65.9)	215/444 (48.4)	154/468 (32.9)	151/685 (22.0)	121/813 (14.9)	110/1172 (9.4)	117/1591 (7.4)	148/3070 (4.8)	190/5954 (3.2)	447/20 732 (2.2)
AOR (95% CI)	0.44 (0.28 to 0.69)	0.68 (0.50 to 0.91)	0.83 (0.65 to 1.04)	0.91 (0.73 to 1.12)	0.98 (0.79 to 1.22)	0.97 (0.79 to 1.19)	0.85 (0.68 to 1.05)	1.00 (0.80 to 1.25)	0.88 (0.71 to 1.10)	0.99 (0.83 to 1.20)	0.92 (0.78 to 1.09)	1.02 (0.88 to 1.17)

Adjusted odds ratios (AOR) and 95% confidence intervals estimated with group not exposed to antenatal corticosteroids used as reference category. Cohort includes infants born in hospital 2009-13 and gestational age 23-34 weeks. Logistic regressions adjusted for birth weight, sex, mode of delivery, and multiple births.

**Figure f2:**
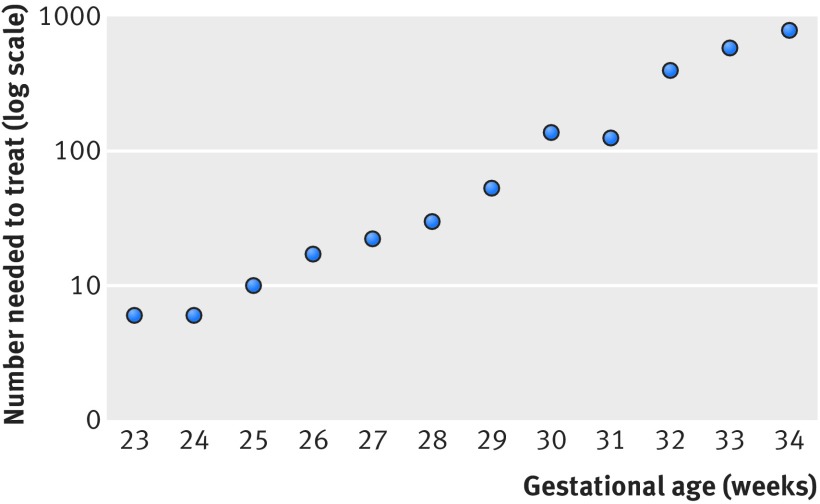
**Fig 2** Number needed to treat with antenatal corticosteroids to prevent one death before discharge in infants with gestational age 23 0/7 to 34 6/7 (logarithmic scale). Number needed to treat to prevent one infant death increased exponentially with increasing gestational age

Exposure to antenatal corticosteroids was associated with lower rates of the composite outcome of death with other common morbidities of prematurity. Infants exposed to antenatal corticosteroids had a lower rate of severe intracranial hemorrhage or death compared with infants without exposure at each gestational age from 23 to 31 weeks (table 2). Infants exposed to antenatal corticosteroids had a lower rate of stage 2 or above necrotizing enterocolitis or death compared with infants without exposure at each gestational age from 23 to 30 weeks. Infants exposed to antenatal corticosteroids also had a lower rate of severe retinopathy of prematurity or death than did infants without exposure at each gestational age from 23 to 29 weeks. The rate of bronchopulmonary dysplasia or death was lower in infants exposed to antenatal corticosteroids than in infants without exposure only among infants at 23 and 24 weeks’ gestation.

We also did similar analyses of non-composite secondary outcomes (table 3). Infants exposed to antenatal corticosteroids had a lower rate of severe intracranial hemorrhage compared with infants without exposure at each gestational age from 23 to 30 weeks. Rates of stage 2 or above necrotizing enterocolitis and severe retinopathy of prematurity were higher in infants exposed to antenatal corticosteroids than in infants without exposure at 23 weeks’ gestation and did not differ among infants at other gestations. Rates of bronchopulmonary dysplasia were higher among infants exposed to antenatal corticosteroids compared with infants without exposure at 23 and 24 weeks’ gestation and did not differ among infants at other gestations. Rates of survival without major hospital morbidity were higher among infants exposed to antenatal corticosteroids compared with those without exposure at 23, 24, 26, and 27 weeks’ gestation and did not differ among infants at other gestations. Infants exposed to antenatal corticosteroids had a lower rate of mechanical ventilation on day 0, 1, and/or 2 after birth compared with infants who were not exposed at each gestational age from 25 to 34 weeks’ gestation and did not differ among infants at other gestations (table 3).

**Table 3 tbl3:** Morbidities of infants born 23 0/7 to 34 6/7 by gestational age and exposure to antenatal corticosteroids (ANS). Values are numbers (percentages) unless stated otherwise

	23 weeks	24 weeks	25 weeks	26 weeks	27 weeks	28 weeks	29 weeks	30 weeks	31 weeks	32 weeks	33 weeks	34 weeks
**Survival without major hospital morbidity**												
ANS	51/754 (6.8)	270/1781 (15.2)	594/2161 (27.5)	1127/2602 (43.3)	1988/3315 (60.0)	3068/4237 (72.4)	4125/5019 (82.2)	5676/6466 (87.8)	7817/8556 (91.4)	12 409/13 203 (94.0)	16 177/16 810 (96.2)	16 507/16 928 (97.5)
No ANS	8/447 (1.8)	38/352 (10.8)	96/381 (25.2)	175/444 (39.4)	265/468 (56.6)	494/685 (72.1)	653/813 (80.3)	1008/1172 (86.0)	1443/1591 (90.7)	2876/3070 (93.7)	5728/5954 (96.2)	20 220/20 732 (97.5)
AOR (95% CI)	4.19 (2.07 to 9.68)	1.64 (1.15 to 2.41)	1.26 (0.98 to 1.64)	1.27 (1.03 to 1.58)	1.23 (1.00 to 1.50)	1.09 (0.90 to 1.31)	1.19 (0.98 to 1.44)	1.18 (0.98 to 1.42)	1.08 (0.89 to 1.30)	1.08 (0.91 to 1.27)	1.04 (0.88 to 1.21)	0.99 (0.87 to 1.13)
**Severe intracranial hemorrhage**												
ANS	186/754 (24.7)	314/1781 (17.6)	267/2161 (12·.4)	204/2602 (7.8)	146/3315 (4.4)	126/4237 (3.0)	76/5019 (1.5)	63/6466 (1.0)	50/8556 (0.6)	34/13 203 (0.3)	25/16 810 (0.1)	12/16 928 (0.1)
No ANS	133/447 (29.8)	92/352 (26.1)	71/381 (18.6)	78/444 (17.6)	47/468 (10.0)	36/685 (5.3)	25/813 (3.1)	26/1172 (2.2)	15/1591 (0.9)	12/3070 (0.4)	10/5954 (0.2)	13/20 732 (0.1)
AOR (95% CI)	0.75 (0.57 to 0.98)	0.61 (0.46 to 0.80)	0.60 (0.45 to 0.81)	0.40 (0.30 to 0.54)	0.40 (0.29 to 0.58)	0.56 (0.38 to 0.83)	0.50 (0.32 to 0.81)	0.44 (0.28 to 0.71)	0.61 (0.35 to 1.12)	0.65 (0.35 to 1.32)	0.87 (0.43 to 1.90)	1.08 (0.48 to 2.40)
**Necrotizing enterocolitis ≥stage 2**												
ANS	55/754 (7.3)	129/1781 (7.2)	165/2161 (7.6)	181/2602 (7.0)	150/3315 (4.5)	159/4237 (3.8)	155/5019 (3.1)	154/6466 (2.4)	139/8556 (1.6)	136/13 203 (1.0)	102/16 810 ((0.6)	45/16 928 (0.3)
No ANS	21/447 (4.7)	27/352 (7.2)	32/381 (8.4)	28/444 (6.5)	32/468 (6.8)	28/685 (4.1)	30/813 (3.7)	37/1172 (3.2)	21/1591 (1.3)	40/3070 (1.3)	28/5954 (0.5)	66/20 732 (0.3)
AOR (95% CI)	1.71 (1.02 to 2.97)	0.92 (0.60 to 1.45)	0.88 (0.59 to 1.34)	1.05 (0.71 to 1.61)	0.65 (0.44 to 0.99)	0.91 (0.61 to 1.42)	0.82 (0.55 to 1.27)	0.71 (0.50 to 1.05)	1.21 (0.78 to 1.97)	0.75 (0.53 to 1.08)	1.25 (0.83 to 1.93)	0.83 (0.56 to 1.22)
**Severe retinopathy of prematurity**												
ANS	104/754 (13.8)	301/1781 (16.9)	279/2161 (12.9)	192/2602 (7.4)	89/3315 (2.7)	62/4237 (1.5)	43/5019 (0.9)	22/6466 (0.3)	17/8556 (0.2)	9/13 203 (0.1)	2/16 810 (0.0)	1/16 928 (0.0)
No ANS	37/447 (8.3)	48/352 (13.6)	49/381 (12.9)	38/444 (8.6)	16/468 (3.4)	10/685 (1.5)	6/813 (0.7)	2/1172 (0.2)	2/1591 (0.1)	0/3070 (0.0)	2/5954 (0.0)	1/20 732 (0.0)
AOR (95% CI)	1.75 (1.18 to 2.65)	1.20 (0.87 to 1.68)	0.92 (0.66 to 1.29)	0.76 (0.53 to 1.12)	0.68 (0.40 to 1.22)	0.84 (0·.44 to 1.76)	1.51 (0.60 to 5.06)	0.79 (0.32 to 2.36)	1.47 (0.42 to 9.31)	1.62 (0.30 to 2.99)	0.47 (0.08 to 3.61)	0.47 (0.02 to 4.93)
**Bronchopulmonary dysplasia**												
ANS	274/754 (36.3)	838/1781 (47.1)	1029/2161 (47.6)	1021/2602 (39.2)	952/3315 (28.7)	850/4237 (20.1)	633/5019 (12.6)	543/6466 (6.2)	514/8556 (6.0)	594/13 203 (4.5)	491/16 810 (2.9)	357/16 928 (2.1)
No ANS	111/447 (24.8)	126/352 (35.8)	159/381 (41.7)	150/444 (33.8)	108/468 (23.1)	110/685 (16.1)	96/813 (11.8)	89/1172 (7.6)	98/1591 (6.2)	130/3070 (4.2)	174/5954 (2.9)	411/20 732 (2.0)
AOR (95% CI)	1.76 (1.35 to 2.31)	1.57 (1.24 to 2.00)	1.23 (0.98 to 1.54)	1.20 (0.96 to 1.49)	1.26 (0.99 to 1.60)	1.19 (0.96 to 1.50)	0.97 (0.77 to 1.23)	1.10 (0.87 to 1.41)	0.96 (0.77 to 1.22)	1.06 (0.87 to 1.29)	0.97 (0.81 to 1.16)	1.08 (0.93 to 1.25)
**Ventilated day 0, 1, and/or 2 after birth**												
ANS	743/754 (98.5)	1728/1781 (97.0)	1974/2161 (91.3)	2108/2602 (81.0)	2259/3315 (68.1)	2432/4237 (57.4)	2227/5019 (44.4)	1991/6466 (30.8)	1872/8556 (21.9)	1872/13 203 (14.2)	1416/16 810 (8.4)	836/16 928 (4.9)
No ANS	434/447 (97.1)	339/352 (96.3)	359/381 (94.2)	410/444 (92.3)	393/468 (84.0)	497/685 (72.6)	492/813 (60.5)	525/1172 (44.8)	537/1591 (33.8)	727/3070 (23.7)	755/5954 (12.7)	1513/20 732 (7.3)
AOR (95% CI)	1.83 (0.79 to 4.32)	1.29 (0.64 to 2.38)	0.51 (0.30 to 0.81)	0.29 (0.19 to 0.42)	0.38 (0.29 to 0.50)	0.48 (0.40 to 0.58)	0.49 (0.42 to 0.58)	0.53 (0.47 to 0.61)	0.55 (0.49 to 0.62)	0.52 (0.48 to 0.58)	0.63 (0.57 to 0.69)	0.67 (0.61 to 0.73)

Adjusted odds ratios (AOR) and 95% confidence intervals estimated with group not exposed to antenatal corticosteroids used as reference category. Cohort includes infants born in hospital 2009-13 and gestational age 23-34 weeks. Logistic regressions adjusted for birth weight, sex, mode of delivery, and multiple births.

## Discussion

This large multicenter prospective cohort study illustrates that exposure to antenatal corticosteroids is associated with lower mortality in infants at most gestational ages from 23 to 34 weeks, even after adjustment for multiple significant confounders. Importantly, this study reports for the first time that the number needed to treat associated with a lower mortality increases exponentially at the higher gestational ages above 24 weeks. Although the adjusted odds ratios were similar at different gestational ages, the number needed to treat decreased among infants at the lowest gestations as the absolute risk reductions were larger among infants at lower gestations. We also found that exposure to antenatal corticosteroids was associated with lower rates of important composite outcomes including severe intracranial hemorrhage or death, stage 2 or above necrotizing enterocolitis or death, and severe retinopathy of prematurity or death at most gestations. We noted that survival without major hospital morbidities was higher among infants at the lowest gestational ages who were exposed to antenatal corticosteroids. Thus, the benefits of antenatal corticosteroids seem to be larger in infants born at lower gestations for which data are most limited.

### Strengths and limitations of study

Although the data used in this study are not population based, this is the largest observational study on antenatal corticosteroid exposure to date and the only one that assessed exposure at all gestational ages at which antenatal corticosteroids are recommended. A large proportion of infants at 23 weeks’ gestation did not receive antenatal corticosteroids compared with most other gestational age groups, and this study may provide a good mimic of the experimental situation at this gestational age in particular. In addition, the large sample size in this study would have improved the precision of the estimated effect size. Data came from both academic and non-academic, as well as large and small, neonatal intensive care units across the US, but some limitations should be noted. An inception cohort of fetuses who were either exposed or not exposed to antenatal corticosteroids was not available. The timing of antenatal corticosteroid administration in relation to gestational age at administration, time from administration to delivery, and whether the course of antenatal corticosteroids was partial, complete, or repeated were not available. However, inclusion of infants who received antenatal corticosteroids outside the optimal window for administration,^20^ from more than 24 hours to seven days, would be expected to skew the results toward the null. Inclusion of infants who received repeated doses of corticosteroids would not be expected to change the rate of hospital mortality or any of the individual major hospital morbidities included in the study significantly compared with a single dose.^14^ ^21^

Furthermore, data were not collected on whether fetal monitoring was undertaken or the length of maternal hospital admission before delivery.^22^ ^23^ The indication for preterm delivery was not available and may differ between groups. Women admitted in advanced labor most likely would be over-represented in the group that did not receive antenatal corticosteroids, and adjustment for this potential bias was not possible. The results of this study are unlikely to be due only to confounding, but some residual unmeasured bias in the results due to baseline differences may exist between the study groups that was not accounted for in the regression models. Multiple testing was used in this study with a 5% significance level, and this may have resulted in a few results being significant purely by chance, although this is unlikely as the benefits seemed to be consistent across different gestations. In addition, there is a possibility of postnatal bias in which infants not exposed to antenatal corticosteroids may have had their care restricted or withheld.^3^ ^24^ ^25^ ^26^

### Comparison with other studies

This study shows a survival benefit associated with exposure to antenatal corticosteroids at most gestational ages from 23 to 34 weeks. In the Cochrane review of randomized controlled trials of administration of antenatal corticosteroids, subgroup analysis of infants delivered at less than 30 weeks’ gestation showed that rates of neonatal death were not significantly decreased by the intervention (relative risk 0.82, 95% confidence interval 0.60 to 1.11; one study, 150 infants).^1^ When examined by gestational age at trial entry rather than gestational age at birth, the mortality rate was lower among infants from 26 weeks and later in this meta-analysis. Only 49 extremely preterm infants less than 26 weeks’ gestation were included in that meta-analysis, and no infants less than 24 weeks’ gestation were included. The benefits among infants at higher gestations in our study were less consistent than the benefits described in the meta-analysis, possibly because these infants have low rates of major adverse outcomes in the current era. We did not consider the effect of antenatal corticosteroid administration among infants beyond 34 weeks’ gestation in our study, which focused on death and major hospital morbidities.

This study supports and extends the results of the academic center based NICHD Neonatal Research Network study of 10 541 infants delivered between 22 and 25 weeks’ gestation, which found that infants exposed to antenatal corticosteroids had a lower rate of death, a lower rate of death or severe intracranial hemorrhage/periventricular leukomalacia, and a lower rate of death or necrotizing enterocolitis at each gestation from 23 to 25 weeks.^7^ Our study adds to the results from an observational study of 11 607 infants from 22 to 33 weeks’ gestation that found a lower mortality rate among infants exposed to antenatal corticosteroids only at 22-27 weeks’ gestation; this was in part a result of the smaller sample size at each gestational age in that study, as the adjusted hazard ratios were substantially less than 1.0 at most gestational ages.^8^ Our study is consistent with and extends the results from a cohort study that included 13 406 infants born between 23 and 32 weeks’ gestation, which showed a lower mortality only in infants born between 24 and 29 weeks gestation exposed to antenatal corticosteroids compared with unexposed infants, but again the sample size was smaller.^9^

In the Cochrane review of randomized controlled trials of administration of antenatal corticosteroids, the subgroup analysis of infants less than 28 weeks’ gestation showed a significant reduction in the rates of intracranial hemorrhage (relative risk 0.34, 0.14 to 0.86; one study, 62 infants).^1^ A further randomized controlled trial also found a lower rate of severe intracranial hemorrhage in infants less than 28 weeks’ gestation exposed to antenatal corticosteroids compared with unexposed infants (1/33 *v* 9/38; P=0.01).^27^ In our study, infants exposed to antenatal corticosteroids similarly had a lower rate of severe intracranial hemorrhage. The Cochrane review of randomized controlled trials did not do subgroup analyses by gestational age for necrotizing enterocolitis, retinopathy of prematurity, or bronchopulmonary dysplasia.^1^

Our study showed that antenatal corticosteroids were associated with lower rates of death and important composite outcomes among infants at most gestational ages, including all gestations less than 30 weeks. This finding was primarily due to a lower rate of death in infants exposed to antenatal corticosteroids, which was only partially offset by a higher rate of non-composite outcomes for necrotizing enterocolitis, severe retinopathy of prematurity, and bronchopulmonary dysplasia among infants at the lowest gestations. These differences are likely explained by competing outcomes whereby the rate of death was higher in infants without exposure to antenatal corticosteroids who did not survive long enough to either develop or be diagnosed as having the morbidity. The higher rate of death in infants without antenatal corticosteroids exposure may also explain much of the differences in reported outcomes in other observational studies of antenatal corticosteroids to date. The higher rate of survival without major hospital morbidities among infants at the lowest gestations exposed to antenatal corticosteroids compared with those without exposure in our study is an important observation that may be due to the clustering of more than one morbidity in the same infant.^28^

Studies on the effect of antenatal corticosteroids on the rates of bronchopulmonary dysplasia have had conflicting results. The traditional definition of bronchopulmonary dysplasia used in this study probably overestimates the incidence of bronchopulmonary dysplasia compared with the physiologic definition used in studies by the NICHD Neonatal Research Network.^7^ ^29^ The aforementioned NICHD Neonatal Research Network study found that infants exposed to antenatal corticosteroids had a lower rate of death or bronchopulmonary dysplasia only at 23 weeks’ gestation. Two other observational studies of antenatal corticosteroid exposure found that infants exposed to antenatal corticosteroids did not have a lower rate of traditional bronchopulmonary dysplasia by gestational age, but these two studies did not control for mortality in their analyses of bronchopulmonary dysplasia.^8^ ^9^ Infants can be diagnosed as having bronchopulmonary dysplasia only if they survive to 36 weeks’ postmenstrual age, and the higher survival rate in this study among infants at the lowest gestations exposed to antenatal corticosteroids may have resulted in more infants being diagnosed as having bronchopulmonary dysplasia.

This study differs from the study by the Neonatal Research Network of Japan, in which results were reported for two week periods rather than single weeks’ of gestation.^8^ That study found no difference in the rate of necrotizing enterocolitis in any gestational age group and found no difference in rates of intracranial hemorrhage in infants from 22 to 23 weeks’ gestation. Our study also differs from the study by the Neonatal Intensive Care Units, which found a higher rate of bronchopulmonary dysplasia in infants delivered at 24 and 26 weeks’ gestation and a higher rate of intracranial hemorrhage in infants at 23 weeks’ gestation exposed to a complete course of antenatal corticosteroids.^10^ The results of the gestational age subgroup analysis in that study may have been affected by the inclusion of infants with partial exposure to antenatal corticosteroids and infants without exposure to antenatal corticosteroids in the same group.^1^

The Cochrane review of randomized controlled trials of administration of antenatal corticosteroids found a decreased rate of respiratory distress syndrome in infants born before 34 weeks’ gestation (relative risk 0.58, 0.47 to 0.72; five studies, 1177 infants) but not before 28 weeks’ gestation (0.79, 0.53 to 1.18; four studies, 102 infants).^1^ Our study analyzed data on whether infants were ventilated on day 0, 1, and/or 2 as a surrogate marker for severe respiratory distress syndrome, as infants with less severe respiratory distress syndrome may be more likely to be managed with less invasive respiratory support.^30^ ^31^ The lower rate of early mechanical ventilation at each gestational age from 25 to 34 weeks indicates amelioration of the severity of respiratory distress syndrome in these infants. Decisions to provide or withhold active treatment may affect outcomes particularly among infants at the lowest gestations from 23 to 24 weeks.^25^ However, our data indicate that no difference existed in the number of infants at the lowest gestations that died without delivery room resuscitation after birth, as there was no difference in the rate of mechanical ventilation on day 0, 1, or 2 among infants at 23 and 24 weeks’ gestation.

This study supports and extends the findings of a recent systematic review and meta-analysis of previously published data from observational studies of antenatal corticosteroid use, which found a lower rate of mortality among infants at 23 weeks’ gestation who received active treatment.^32^ However, our study adds the seminal finding that the number needed to treat with antenatal corticosteroids to prevent one death before discharge increased from six at 23 and 24 weeks’ gestation to 798 at 34 weeks’ gestation.

### Conclusion and policy implications

The effect on mortality and survival without major morbidities of exposure to antenatal corticosteroids seems to be largest in infants at the lowest gestations, including infants at 23 weeks’ gestation who were not included in randomized controlled trials. The study also found that antenatal exposure to corticosteroids was associated with lower rates of mortality and major hospital morbidities at most gestations for which steroids are currently recommended. This study supports the administration of antenatal corticosteroids in women with threatened preterm labor from 23 to 34 weeks’ gestation.

**What is already known on this topic**

Guidelines recommend administration of antenatal corticosteroids to infants from 24 to 34 weeks’ gestation and consideration of administration at 23 weeks’ gestation
The extension to consider administration at 23 weeks is based on limited data from observational studies and consensus
Meta-analyses of randomized controlled trials of antenatal corticosteroids have shown no difference in mortality in the subgroup analysis of infants delivered at 24-30 weeks’ gestation

**What this study adds**

This study highlights for the first time that infants at the lowest gestations seem to benefit the most from exposure to antenatal corticosteroids
Exposure to antenatal corticosteroids was associated with a comparably lower mortality at 23 weeks’ gestation than at 24 weeks
Survival without major hospital morbidity was also lower among infants exposed to antenatal corticosteroids at the lowest gestations

## Notes

Ethical approval: The study protocol was approved by the Institutional Review Board at the University of Alabama at Birmingham (protocol number N141125009) and by the Research Advisory Committee at MEDNAX, Inc.

Data sharing: No additional data available.

Transparency: The lead author affirms that the manuscript is an honest, accurate, and transparent account of the study being reported; that no important aspects of the study have been omitted; and that any discrepancies from the study as planned (and, if relevant, registered) have been explained.
